# Influence of Protein Intake during Obesity Reduction on Physical Function and Body Habitus in Older Males with Prediabetes: A Pilot Study

**DOI:** 10.1016/j.cdnut.2026.109460

**Published:** 2026-07-25

**Authors:** Connie W Bales, Marshall G Miller, Shelley R McDonald, Jessica T Wallis, Leigh Ann Yeager, Richard J Sloane, Kathryn N Porter Starr

**Affiliations:** 1 GRECC, Durham VA Health Care System, Durham, NC, United States; 2 Department of Medicine, Duke University Medical Center, Durham, NC, United States; 3 Center for the Study of Aging, School of Medicine, Duke University Medical Center, Durham, NC, United States

**Keywords:** obesity intervention, older adults, prediabetes, protein, function

## Abstract

**Background:**

Weight loss can improve health and function in older adults with obesity but may also accelerate loss of fat-free mass. Older men are underrepresented in obesity intervention trials; thus we tested higher protein intake plus exercise to help preserve muscle mass and physical function during weight loss in men with obesity and prediabetes.

**Objectives:**

We evaluated the effects of an enhanced protein weight loss intervention relative to a recommended dietary allowance (RDA)–level protein intervention in older males (veterans) with prediabetes and obesity.

**Methods:**

This study was a randomized controlled trial. Equal numbers of participants, males aged >55 y with obesity [body mass index (BMI), in kg/m^2^> 30] and prediabetes, were randomly assigned to either an RDA protein (0.8 g/kg/d; *n* = 32) or an enhanced protein (1.4 g protein/kg/d with 30 g high-quality protein 4 times/d; *n* = 32) hypocaloric diet plus exercise intervention, with outcome assessments at 3 and 6 mo., namely, Short Physical Performance Battery (SPPB) and fat-free mass (FFM) measured by Bodpod, body weight, body fat, waist circumference, 6-min walk time test (6MWT), 8-foot up-and-go test, and handgrip strength.

**Results:**

Baseline characteristics were as follows: BMI = 35.8 ± 4.3, mean age 67.6 ± 7.0 y, and hemoglobin A1c 6.0% ± 0.3%. For the SPPB score, the Enhanced Protein group had a greater benefit at both 3 mo (small Cohen’s *d* = 0.23) and at 6 mo (moderate Cohen’s *d* = 0.68). The Enhanced Protein group showed an increase in SPPB at 3 mo of 1.5 ± 0.2 units, compared with 1.2 ± 0.3 units for the RDA Protein group. The amount of weight lost was also more in the Enhanced Protein group at 3 mo (4.7% ± 0.9 compared with 3.1% ± 0.7). Additionally, participants in the Enhanced Protein group preserved more FFM at 6 mo (moderate group effect, Cohen’s *d* = 0.48; 95% confidence interval [CI]: 0.26, 0.62), with a loss of 0.8 ± 0.3 kg compared with the RDA Protein arm, who lost 1.5 ± 0.4 kg. Other Enhanced Protein group effects at 3 mo included an increased distance walked in the 6MWT by 63.3 ± 12.8 m [mean difference (MD): 29.0 m; *d* = 0.53; 95% CI: <0, 1.19] and increased handgrip strength by 3.7 ± 1.2 kg (MD: −2.8 kg; *d* = 0.56; 95% CI: 0.21, 0.88), relative to the RDA Protein group, which had increases of 34.4 ± 11.9 m and 0.8 ± 1.0 kg for 6MWT and handgrip strength, respectively. The handgrip effect remained at 6 mo for Enhanced Protein, with an increase of 3.0 ± 1.5 kg compared with an increase of 1.2 ± 1.4 kg in RDA Protein (MD: −1.8; *d* = 0.30; 95% CI: 0.15, 0.35).

**Conclusions:**

The results provide preliminary evidence that an enhanced protein regimen can benefit older males with prediabetes, especially with regard to physical function and body composition.

This trial was registered at clinicaltrials.gov as NCT03835416.

## Introduction

Nearly 40% of adults aged 65 y and older have a BMI (in kg/m^2^) in the obesity range (≥30), underscoring the growing burden of excess adiposity in later life [1]. In this population, obesity poses a particular threat because it exacerbates a host of age-related chronic conditions, including diabetes, heart disease, hypertension, stroke, and certain cancers. It also promotes osteoarthritis and accelerates age-related functional decline [2]. Obesity is the leading risk factor for developing type 2 diabetes. It thus poses a critical threat to physical function in older adults because both obesity and diabetes have direct detrimental effects on muscle health [3]. Excessive adipose stores contribute to a persistent low-grade state of inflammation, leading to muscle depletion by enhancing protein breakdown and impairing myogenesis [4]. Excessive adiposity also promotes lipid accumulation between and within muscles, reducing muscle quality [3,5]. Although the direct mechanism of diabetic myopathy is not fully understood, accelerated sarcopenia and dynapenia are common in older adults with long-term type 2 diabetes [6]. Up to 70% of those with diabetes have lower extremity mobility limitations [7,8].

Obesity reduction in older adults has been shown to improve health and function; it markedly slows progression of prediabetes into type 2 diabetes [8]. This is important because, without intervention, individuals with prediabetes have a 15% to 30% chance of progressing into type 2 diabetes within 5 y [2]. Along with a host of other health benefits, obesity reduction has been demonstrated to improve physical function in older adults [9,10]. Yet, weight reduction may be challenging for older adults, who typically have lower caloric requirements owing to a reduced metabolic rate and may have reduced levels of physical activity and increased prevalence of comorbidities that complicate diet behavior modification. In older males, obesity reduction is understudied, as older females tend to be better represented in clinical trials [11]. Moreover, there are relatively few studies of obesity reduction in older Black individuals, even though obesity rates are exceptionally high in the Black community [12].

An important concern related to obesity reduction in older adults is the potential negative impact on physical function because of the loss of fat-free mass (FFM), which occurs concomitantly with the loss of fat mass [13]. With conventional weight loss regimens, the loss of FFM can constitute 25% or more of the total amount of weight lost [13]. Such substantial loss of FFM is clearly undesirable in older adults who are already at risk of sarcopenia. Reduced FFM brings elevated risk for those with prediabetes or type 2 diabetes, not only for their functional status but also because reduced muscle mass is linked with impaired glucose uptake and insulin resistance [8]. These and other concerns have prompted the study of obesity intervention protocols for older adults that are aimed at delaying or preventing loss of FFM. The main avenues for preserving/promoting muscle protein anabolism and potentially mitigating the loss of FFM during the challenge of calorie restriction are *1*) adequate/generous high-quality protein intake [14] and *2*) resistance exercise training [15]. However, age-related anabolic resistance, that is, resistance to the anabolic effects of protein ingestion, presents a barrier to this approach in older individuals. To overcome this barrier, our team [10] and others [16] have explored interventions using higher protein weight-loss diets to preserve FFM and physical function during obesity reduction in older adults. This approach is based on short-term studies showing that essential amino acids, especially leucine, initiate the mechanistic target of rapamycin signaling pathway to stimulate muscle protein synthesis [[17], [18], [19]] and that a balanced intake of ∼30 g of protein at each meal is needed for optimal protein synthesis in the aging muscle [20,21]. Our team found that, in older adults with obesity and functional impairment, higher protein intakes (30 g high-quality protein at each of 3 meals) in combination with a hypocaloric diet resulted in clinically meaningful improvements in physical performance [10]. The benefits of higher protein diets for older adults continue to be recognized and recommended [16,22], albeit with some equivocal findings in the literature to date [22], likely owing to factors including variability in protein dose and duration and extent of overweight/obesity at baseline.

In the case of the prediabetes/diabetes population, there is an additional gap in the evidence with regard to the impact of higher protein intake on glycemic control. One trial has suggested that a higher protein intake might blunt benefits to insulin sensitivity typically accrued from weight loss [23], and the authors noted that acute protein ingestion has been shown to reduce insulin sensitivity [24]. However, 2 randomized controlled trials (RCTs) have demonstrated a beneficial effect of higher protein intakes on insulin sensitivity [25,26]. These contradictory observations underscore the critical need for controlled trials exploring the impact of higher protein diets in high-risk populations who stand to benefit most from this research, namely, older adults with obesity who are at risk of type 2 diabetes and, in particular, Black individuals. For older individuals with obesity and insulin resistance, the benefits of a higher protein intake during weight reduction could be substantial, circumventing loss of muscle mass while slowing the progression of prediabetes. Recognizing the dearth of evidence on this topic, we designed this pilot trial to assess changes in physical function [measured by Short Physical Performance Battery (SPPB)] and FFM, along with other measures of body composition and physical function, in Black and White males with obesity and prediabetes.

## Methods

### Study design, population, and enrollment

This was a randomized, controlled, parallel-group design with repeated measures. Study design and implementation followed CONSORT guidelines. The institutional review boards of the Durham Veterans Affairs Medical Center (VAMC) and the Duke University School of Medicine reviewed and approved this study. The trial was registered in a public registry (ClinicalTrials.gov identifier: NCT03835416), and the study nickname is VALOR-UP, "Veterans Achieving Weight Loss and Optimizing Resilience-Using Protein.” Following baseline assessment, participants meeting inclusion criteria were randomly assigned to 1 of 2 study arms (1:1 allocation ratio) using block randomization to stratify by race (White, Black) for a 6-mo intervention:

• Recommended dietary allowance (RDA) Protein: 0.8 g protein per kg body weight/d.
• Enhanced Protein: ≥30 g of high-quality protein per meal, 1.4 g protein per kg body weight/d.

#### Participants

English-speaking male veterans, of Black or White race, aged ≥55 y, with BMI ≥30 and prediabetes, defined as fasting blood glucose (FBG) of ≥95 and <126 mg/dL or hemoglobin A1c (HbA1c, glycated hemoglobin) in the range of 5.7% to 6.4%, were enrolled. Participants needed to be able to walk independently (cane/walker permitted), be able to participate in a low-intensity exercise program, and have an SPPB score of 4 to 11 out of 12. Exclusions at screening included glomerular filtration rate (GFR) <45 mL/min/1.73 m^2^, the presence of a neurological condition affecting function that would not be expected to be changed by the intervention (e.g., Parkinson disease, multiple sclerosis), use of non-oral medications to control blood sugar, self-report of diabetes, or notation of a diabetes diagnosis in the medical record by the study physician. At the beginning of the trial (December 2019), use of any diabetes medication was exclusionary, but in October 2022, the protocol was modified to include oral diabetes medication because of changes in the management of prediabetes at our medical center, leading to the prescription of these medicines. From this point, sulfonylureas, biguanides, thiazolidinediones, α-glucosidase inhibitors, meglitinides, dipeptidyl peptidase-4 inhibitors, sodium-glucose cotransporter-2 inhibitors, bile acid sequestrants, and dopamine agonists were permitted if being taken consistently for ≥3 mo.

#### Recruitment and enrollment

To obtain a list of veterans who might potentially qualify for study participation, we used the United States Department of Veterans Affairs (VA) Informatics and Computing Infrastructure to conduct queries of electronic medical records to identify veterans who had been seen at the Durham VAMC in the past year, were aged ≥55 y, and had obesity (BMI ≥ 30) and prediabetes as defined above. These participants received a letter describing the study and were given the option to opt out of being contacted. Those who did not opt out were contacted by telephone and, when reached and willing, participated in a telephone screening interview. We also recruited participants who were referred to the MOVE Program at the Durham VAMC. MOVE is a weight management and health promotion program designed to improve the lives of veterans [27]. Before starting the MOVE program, veterans who were recently referred and attending the MOVE orientation were given the option to delay joining the MOVE program to participate in the research study. We also recruited veterans who were graduates of the MOVE program but remained study eligible based on enrollment criteria.

Initial study recruitment and enrollment began in late 2019, but in March 2020, in-person visits were suspended at our medical centers for all nonessential clinical research activities because of the COVID-19 pandemic. For the 10 participants already enrolled, we quickly transitioned to virtual and contactless delivery of the interventions, and 8 of them were able to continue participation for the full 6 mo. However, some midpoint and end point outcome data for these participants could not be collected because of continued restrictions on in-person contact, and we were unable to enroll any new participants during that time interval. For these 8 participants, in-person measures were not collected at midpoint and endpoint for SPPB, BodPod, waist circumference (WC), 6-min walk test (6MWT), 8-foot up-and-go test (8UG), and handgrip; body weights were by self-report. Study enrollment, with full collection of all outcomes, resumed in October 2021.

### Intervention protocols

#### Hypocaloric diet

For participants in both study arms, calorie prescriptions were calculated to promote weight loss at a rate of 1 to 2 pounds (mean of 0.6 kg) per week by decreasing total energy intake by ∼500 to 1000 kcal/d. Initial prescribed calorie ranges were derived from calculations of estimated total energy expenditure using equations published by the Institute of Medicine (Washington, DC, USA) based on weight, height, gender, age, and activity coefficient [28]. The sedentary activity coefficient was used as a conservative starting assumption, and calorie prescriptions were subsequently adjusted based on observed weight loss trajectory and participant dietary intake reports. Each participant met individually with an intervention dietitian for training on constructing a meal plan for the prescribed number of servings from each food group per day to achieve protein and calorie intake goals. To determine adherence to the prescribed meal plan, participants kept daily food logs, which were reviewed weekly by the registered dietitian interventionists, and feedback was provided to participants in real time.

#### RDA Protein arm

For the RDA Protein group, protein intake followed the recommended dietary allowance (RDA) of 0.8 g/kg body weight, with a macronutrient distribution of 15% protein, 30% fat, and 55% carbohydrates. Participants were provided 7 servings of whey protein powder (15 g serving) per week to support diet affordability. Daily food logs were reviewed weekly by the registered dietitian interventionists to assess adherence to the daily serving of whey protein powder, as well as adherence to the prescribed daily protein intake (0.8 g/kg body weight/d).

#### Enhanced Protein arm

For the Enhanced Protein group, the prescribed protein intake was 1.4 g/kg body weight, with a macronutrient distribution of 30% protein, 30% fat, and 40% carbohydrates. Twenty-one servings of high-quality (≥30 g/serving) protein (namely, lean meats, whey protein powder, and Ensure Max protein drinks) were provided to participants each week to increase compliance, along with instructions for safe handling and storage practices for these foods. Daily food logs were reviewed weekly by the registered dietitian interventionists to assess intake of the provided protein sources, as well as adherence to the prescribed protein intake per meal and daily protein intake (1.4 g/kg body weight/d). Diet prescriptions were calculated so that the calories being prescribed to the Enhanced Protein participants (per height, weight, weight loss goal) were equivalent to the calories prescribed to the RDA Protein group. Interventionists provided counseling on different methods for including the provided proteins as a part of the prescribed meal plans. Interventionists offered guidance for choosing other high-quality protein options to achieve the ≥30 g protein at one of the meals each day.

#### Nutrition and exercise classes

Beginning with the third week after randomization, participants began a weekly group nutrition education class with other participants in their group (RDA Protein or Enhanced Protein). The classes covered diet-related topics along with stress management, behavioral changes necessary for success during the study period, and goal setting. Also, at this time, all participants were prescribed three 30-min bouts of low-intensity exercise per week. One of the 3 bouts was provided as an in-person (or synchronous virtual) low-intensity group exercise class, led by interventionists who were certified group fitness instructors. These sessions utilized resistance bands and other techniques to improve strength, flexibility, and balance; exercises could be completed either while standing or seated. Participants were asked to perform 2 additional 30-min bouts of exercise on their own each week and were provided additional guidance as needed to achieve that goal. We used attendance at group sessions of exercise, along with nutrition class attendance, as measures of adherence. Exercise sessions performed at home were not used for adherence.

### Baseline and outcome measurements

Outcomes were measured by trained research staff using our standardized methods as described below. Demographic and clinical characteristics were collected at baseline by participant report via questionnaires. Whenever possible, staff who conducted assessments were blinded to participants’ randomization assignment, but because of the nature of the study, neither participants nor researchers could be blinded to treatment allocation for some of the outcome and adherence assessments. To reduce potential bias, both the researcher and the participant were blinded to previous results on testing, and detailed assessment protocols and checklists were followed to standardize testing procedures and ensure complete data collection. The SPPB and FFM (in kilograms) were the primary outcomes in the analyses.

#### Physical function measures

The SPPB [29] includes measures of balance (10-s stand in side-by-side, semitandem, and tandem stances), strength (timed chair stands) [30], and gait (timed 4-m walk). Additional physical function measures included the 6MWT that measures the distance walked (m) in 6 min, and the 8UG that measures the time (s) it takes to get up from a chair, walk 8 feet around a cone, and return back to a seated position. Isometric hand grip strength (kg) was measured using a Jamar hydraulic hand dynamometer (Patterson Medical). The participants were tested separately for both the dominant and nondominant hand twice each, with the higher value recorded as the result.

#### Body composition measures

Height (cm) was measured at baseline using a stadiometer. Body weight, fat mass, FFM (all in kg), percent fat mass, and percent FFM were measured at 0, 3, and 6 mo using the air displacement plethysmography method (BodPod; COSMED, Inc); participants fasted for the BodPod measurement, which was conducted while the participants wore spandex clothing and a swim cap. WC (cm; at minimal waist, using Gulick II tape measure) was also measured at these time points.

#### Glycemic control

Fasted blood samples were collected from the arm by venipuncture at 0, 3, and 6 mo. HbA1c was measured as part of the metabolic screening panel by Labcorp.

### Data and statistical analysis

The VALOR-UP trial evaluated meal-based enhancement of protein intake during weight loss by comparing change from baseline in the primary outcome measures (SPPB and FFM) and the set of secondary outcome measures for the Enhanced Protein compared with the RDA Protein group at 3 and 6 mo. The interim measurement (3 mo) was included to provide a more precise assessment of the trajectory of compliance by change in the 2 primary outcomes of function (SPPB) and FFM. Data were double-entered with discrepancies adjudicated. Treatment codes were revealed only after locking of the database at trial’s end. We did not impute missing variables for participants who had only baseline assessments.

At the outset, we intended to test the primary and secondary outcome measures using ordinary least squares (OLS) linear regression at the 3- and 6-mo follow-up assessment time points, adjusting for baseline. As described earlier (in the Methods section), owing to an extensive period of shutdown in clinical research activities during the COVID-19 pandemic, we were unable to reach the target enrollment goal. As a result, we were insufficiently powered to detect group differences over time for the primary and secondary outcome measures using baseline-adjusted OLS and used a different analytic approach. Given the limited sample size, we estimated the effects of treatment groups rather than relying on *P* values for inference. However, the evidence base led us to expect that membership in the Enhanced Protein group would lead to improved associations with indicators of function and body composition. The between-group differences at 3 and 6 mo from baseline [mean difference (MD) and 95% confidence interval (CI)] were calculated for all outcome measures. The Cohen’s *d* effect sizes were calculated by dividing the MD by the pooled SD and were interpreted as *d* = 0.20 (small), *d* = 0.50 (moderate), and *d* = 0.80 (large); 95% CIs were also calculated for Cohen’s *d.* Data analysis was performed using PASW Statistics, version 18.0 (SPSS Inc., Chicago, IL, USA) and SAS, version 9.4 (SAS Institute Inc., Cary, NC, USA).

## Results

### Enrollment and baseline characteristics

**FIGURE 1:**
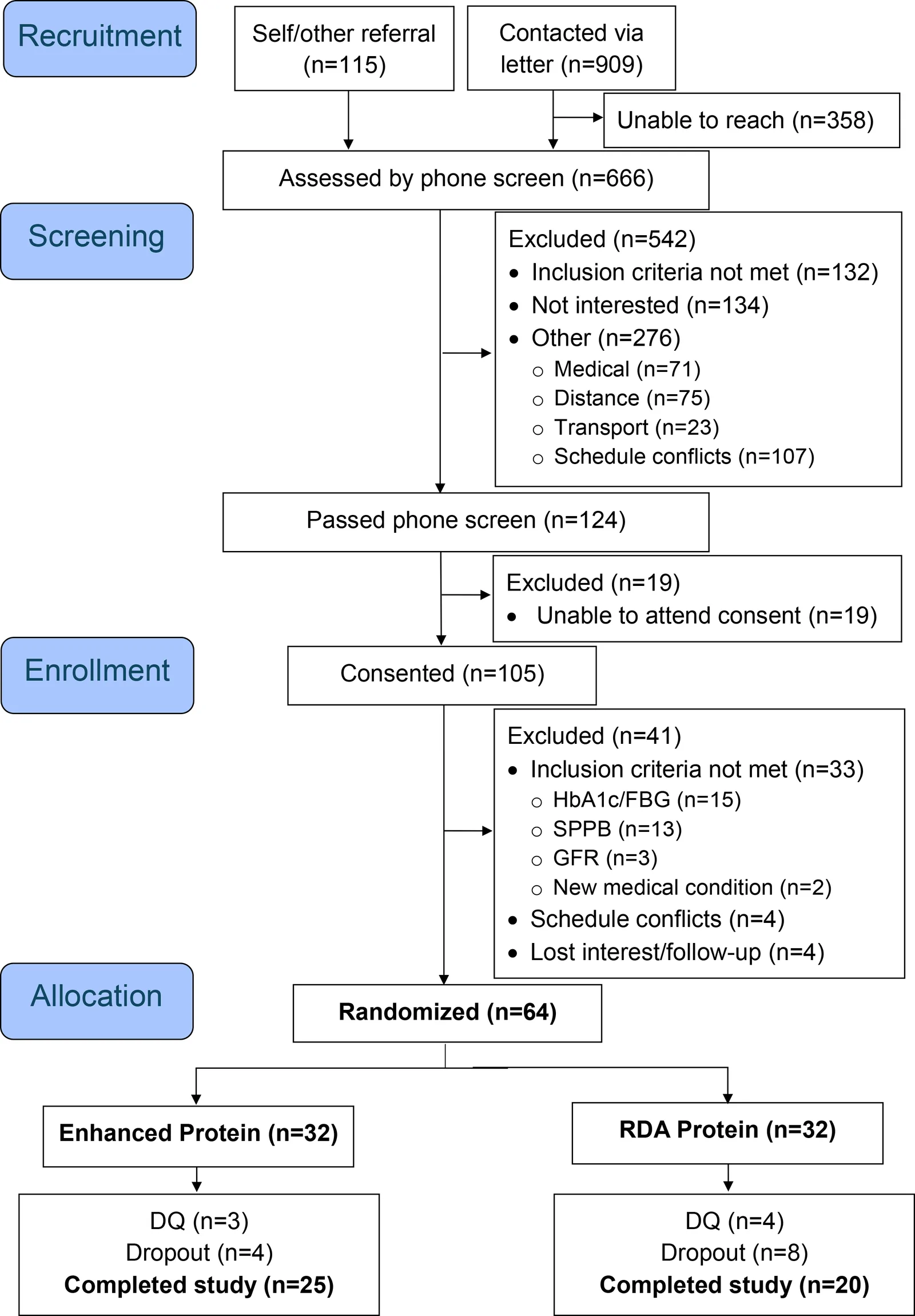
Consort chart of participant involvement in the study, DQ, Disqualified; FBG, fasting blood glucose GFR, glomerular filtration rate; HbA1c, glycated hemoglobin; RDA, recommended dietary allowance; SPPB, short physical performance battery.

A total of 909 veterans were mailed study information letters, and another 115 veterans were recruited through the VA’s MOVE obesity treatment program or by self-referral. Of the 1024 veterans contacted, 666 completed the telephone screening (Figure 1), with 105 prospective participants being consented into the study. At the baseline (in-person) assessment, an additional 41 participants were excluded, mainly for not meeting inclusion criteria (FBG or HbA1c: *n* = 15; SPPB score: *n* = 13; and GFR: *n* = 3) and newly developed medical concerns (*n* = 2). Eight participants had schedule conflicts (*n* = 4), lost interest (*n* = 3), or were lost to follow-up (*n* = 1). A total of 64 participants met all inclusion criteria and were randomly assigned to either the RDA Protein (*n* = 32) or the Enhanced Protein (*n* = 32) arm. Baseline characteristics of the 64 enrolled participants are shown in Table 1. Each arm was equally distributed between White and Black race due to the stratified randomization design. The participants had class II obesity (mean ± SD BMI: 35.8 ± 4.3), a mean ± SD age of 67.6 ± 7.0 y, and a mean ± SD HbA1c of 6.0% ± 0.3%.

**TABLE 1:** Baseline characteristics by diet group

Characteristic	All	RDA Protein	Enhanced Protein
*N*	64	32	32
Age (y),^,^ mean (SD)	67.6 (7.0)	69.2 (7.0)	65.9 (6.9)
Race, *n*			
Black	32	16	16
White	32	16	16
BMI, kg/m^2^, mean (SD)	35.8 (4.3)	35.0 (4.2)	36.5 (4.3)
HbA_1_c, %, mean (SD)	5.9 (0.3)	5.9 (0.3)	5.9 (0.3)

Abbreviations: HbA_1_c, glycated hemoglobin; RDA, recommended dietary allowance.

### Retention and adherence

The rate of dropouts/disqualifications was 30% overall, with a rate of 22% in the Enhanced Protein arm and 38% in the RDA Protein arm (non significant group effect). For the 4 dropouts from the Enhanced Protein group, reasons were unrelated death (1), no longer interested (2), and unknown reasons (1). For the 8 RDA Protein dropouts, reasons were no longer interested (3), study difficulties or time constraints (2), medical issues (1), and unknown reasons (2). Participants were disqualified (*n* = 7) if they developed an exclusion criterion (as listed in the Methods section) after enrollment. Three participants in the Enhanced Protein group were disqualified; the reasons were all physical conditions that would prevent study participation: *1*) new diagnosis of bladder cancer, *2*) development of severe back pain, and *3*) ankle fracture. For the 4 disqualifications in the RDA Protein group, 2 participants became unwilling to complete some study activities and were withdrawn by the principal investigator, and 2 developed physical exclusions, namely, new debilitating pain and unplanned surgery plus complications. Analysis of baseline characteristics for those who dropped out showed no differences from those who did not, except for a borderline (*P* < 0.06) finding of a higher dropout rate for Black compared with White participants.

For participants completing the study, weekly intervention attendance rates for the diet support group classes were 84% for the RDA Protein group and 86% for the Enhanced Protein group. Attendance rates for exercise classes were 83% for the RDA Protein group and 86% for the Enhanced Protein group. Although the intervention duration was 6 mo for most participants, it was 3 mo for a subset of participants (*n* = 5; 3 in RDA Protein, 2 in Enhanced Protein) who enrolled when there were fewer than 6 mo left to go before study funding expired. Those participants who completed their 3-mo participation goal (*n* = 5) were counted as study completers for adherence calculations, but for statistical analyses, their outcomes were included with all the other 3-mo outcome data.

### Intervention results

#### Body weight

**FIGURE 2:**
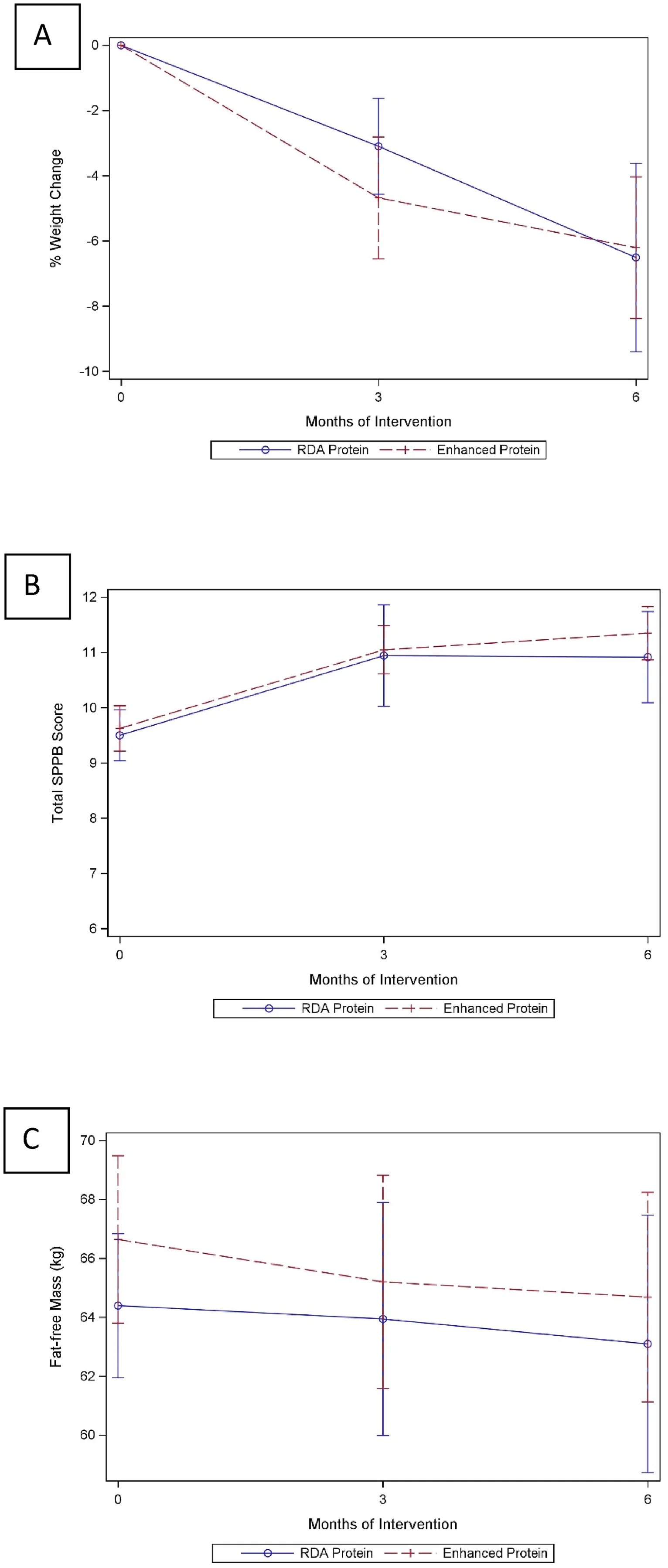
(A) Illustration of the moderate effects on percent change in body weight [means ± 95% confidence interval (CI)] at 3 mo (*d* = 0.43, 95% CI: 0.16, 0.8) for the Enhanced Protein, compared with the recommended dietary allowance (RDA) Protein group. Percent weight losses (mean ± SEM) were 4.7% ± 0.9% for Enhanced Protein and 3.1% ± 0.7% for the RDA Protein group. At 6 mo, there was no group difference in body weight loss by percent (*d* = 0.07, 95% CI: <0, 0.61). (B) Results for the 2 treatment groups for Short Physical Performance Battery (SPPB) (means ± 95% CI) at 3 mo [mean difference (MD) = −0.2 units, *d* = 0.23 (95% CI: <0, 0.42)] and at 6 mo [MD = −0.8 units, *d* = 0.68 (95% CI: 0.41, 0.88)]. The Enhanced Protein SPPB increase (mean ± SEM) at 3 mo was 1.5 ± 0.2 units, compared with 1.2 ± 0.3 units for the RDA Protein group. At 6 mo, the increase for Enhanced Protein was 1.8 ± 0.3 units, compared with 1.0 ± 0.4 for the RDA Protein group. (C) Changes in fat-free mass (FFM) in kg (means ± 95% CI) during weight loss. A small beneficial difference was observed at 3 mo, with (mean ± SEM) 0.7 ± 0.3 kg lost in the Enhanced Protein group compared with 0.9 ± 0.3 kg lost in the RDA Protein arm [MD = −0.2 kg, *d* = 0.15 (95% CI: <0, 0.33)]. At 6 mo, there was a moderate group effect; the Enhanced Protein group lost 0.8 ± 0.3 kg of FFM compared with the RDA Protein group loss of 1.5 ± 0.4 kg [MD = −0.7 kg, *d* = 0.48 (95% CI: 0.26, 0.62)].

As illustrated in Figure 2A and explained in Table 2, moderate effects on body weight, both in kilograms lost (*d* = 0.49; 95% CI: <0, 1.14) and percent change in body weight (*d* = 0.43; 95% CI: 0.16, 0.8), were noted at 3 mo for the Enhanced Protein, compared with the RDA Protein group. At this time point, absolute and percent weight losses were 5.4 ± 1.1 kg and 4.7% ± 0.9%, respectively, for the Enhanced Protein and 3.3 ± 0.8 kg and 3.1% ± 0.7%, respectively, for the RDA Protein group. At 6 mo, there was a small difference (*d* = 0.20; 95% CI: <0, 0.57) by group for weight loss in kilograms (losses were 7.2 ± 1.2 kg for Enhanced Protein and 7.0 ± 1.5 kg for RDA Protein); there was no group difference in body weight loss by percent (*d* = 0.07; 95% CI: <0. 0.61) at 6 mo.

**TABLE 2:** Baseline means and group comparisons at 3 and 6 mo for primary and secondary outcomes

	Baseline mean (95% CI)	3 mo mean^1^ (95% CI)	3 mo mean difference (95% CI)	3 mo Cohen’s d^2^ (95% CI of effect size)	6 mo mean^3^ (95% CI)	6 mo mean difference (95% CI)	6 mo Cohen’s *d* (95% CI of effect size)
Primary outcomes							
SPPB (total units)							
RDA Protein	9.5 (9.0, 10.0)	10.9 (10.0, 11.9)	−0.2 (−1.0 to 0.5)	0.23 (<0 ,0.42)	10.9 (10.1, 11.8)	−0.8 (−1.8 to 0.1)	0.68 (0.41, 0.88)
Enhanced Protein	9.6 (9.2, 10.0)	11.1 (10.6, 11.5)			11.4 (10.9, 11.8)		
Fat-free mass (kg)							
RDA Protein	64.4 (61.9, 66.9)	63.9 (60.0, 67.9)	−0.2 (−.1 to 0.7)	0.15 (<0, 0.33)	63.1 (58.7, 67.5)	−0.7 (−1.7 to 0.4)	0.48 (0.26, 0.62)
Enhanced Protein	66.6 (63.8, 69.5)	65.2 (61.6, 68.8)			64.7 (61.1, 68.3)		
Secondary outcomes							
Body weight (kg)							
RDA Protein	108.5 (104.1, 112.8)	104.4 (97.8, 110.9)	2.1 (−0.7 to 4.8)	0.49 (<0, 1.14)	98.8 (91.7, 105.8)	0.1 (−3.9 to 4.2)	0.20 (<0, 0.57)
Enhanced Protein	113.4 (107.2, 119.6)	107.1 (99.9, 114.4)			105.3 (97.9, 112.7)		
Body weight (%)							
RDA Protein	0	−3.1 (−4.6, −1.6)	1.6 (−0.8 to 4.0)	0.43 (0.16, 0.80)	−4.7 (−6.6, −2.8)	−0.3 (−2.7 to 3.1)	0.07 (<0, 0.61)
Enhanced Protein	0	−4.7 (−6.6, −2.8)			−6.2 (−8.4, −4.0)		
Body fat (kg)							
RDA Protein	44.3 (40.7, 47.9)	40.4 (35.7, 45.1)	2.3 (−0.1 to 4.6)	0.62 (0.03, 1.27)	35.7 (29.7, 41.7)	0.8 (−2.7 to 4.3)	0.17 (<0, 0.64)
Enhanced Protein	46.7 (42.6, 50.9)	41.9 (37.1, 46.8)			40.6 (35.6, 45.6)		
Body fat (%)							
RDA Protein	40.5 (38.5, 42.4)	38.5 (35.7, 41.3)	1.3 (−0.1 to 2.6)	0.62 (0.33, 1.26)	35.8 (31.6, 40.0)	0.4 (−1.8 to 2.6)	0.13 (<0, 0.81)
Enhanced Protein	40.8 (39.0, 42.6)	38.8 (36.3, 41.2)			38.1 (35.5, 40.7)		
Fat-free mass (%)							
RDA Protein	59.5 (57.6, 61.5)	61.5 (58.7, 64.3)	−1.3 (−2.6 to 0.1)	0.62 (0.33, 1.26)	64.2 (60.0, 68.4)	−0.4 (−2.5 to 1.8)	0.13 (<0, 0.81)
Enhanced Protein	59.2 (57.4, 61.0)	61.2 (58.8, 63.7)			61.9 (59.3, 64.5)		
Waist circumference (cm)							
RDA Protein	115.0 (112.0, 118.0)	111.9 (107.4, 116.4)	1.5 (−1.0 to 4.0)	0.38 (0.06, 0.60)	107.7 (102.3, 113.2)	−0.1 (−3.6 to 3.3)	0.03 (<0, 0.72)
Enhanced Protein	115.6 (112.2, 119.0)	111.8 (107.2, 116.5)			111.5 (106.9, 116.0)		
6 Min walk (m)							
RDA Protein	469.0 (428.4, 509.6)	497.6 (438.8, 556.5)	29.0 (−65.1 to 7.2)	0.53 (<0, 1.19)	543.3 (483.8, 602.8)	2.2 (−38.4 to 42.8)	0.04 (<0, 0.78)
Enhanced Protein	483.1 (449.6, 516.6)	540.3 (496.9, 583.6)			533.9 (488.2, 579.7)		
8UG (s)							
RDA Protein	8.1 (7.2, 8.9 )	7.0 (6.0, 8.1)	0.3 (−0.8 to 1.4)	0.18 (<0, 0.84)	6.5 (5.3, 7.8)	0.0 (−1.4 to 1.4)	0.01 (<0, 0.76)
Enhanced Protein	7.9 (7.1, 8.7)	6.6 (6.2, 7.0)			6.4 (5.9, 6.9)		
Handgrip (kg)							
RDA Protein	42.9 (39.1, 46.6)	44.2 (39.0, 49.4)	−2.8 (−6.1 to 0.4)	0.56 (0.21,0.88)	45.1 (38.6, 51.6)	−1.8 (−6.3 to 2.7)	0.30 (0.15, 0.45)
Enhanced Protein	43.9 (39.8, 48.0)	47.2 (42.6, 51.8)			45.6 (41.0, 50.2)		

CI, confidence interval; RDA, recommended dietary allowance; SPPB, Short Physical Performance Battery; 8UG, 8 foot up and go test.

1 Number of participants was 18 for RDA Protein and 21 for Enhanced Protein, with the exception that it was 17 for RDA Protein for 6 min walk and 8UG.

2 Cohen’s *d* effect sizes interpreted as *d* = 0.20 (small), *d* = 0.50 (moderate) and *d* = 0.80 (large). 95% Lower confidence intervals <0 reflects negative values.

3 Number of participants was 12 for RDA Protein and 20 for Enhanced Protein.

#### Primary outcomes

Figures 2B and 2C present the findings for SPPB and FFM, respectively; between-group effect sizes (Cohen’s *d* and 95% CI) for these outcomes are shown in Table 2. For physical function defined as the SPPB total score, there was a small effect at 3 mo (MD: −0.2 units; *d* = 0.23; 95% CI: <0.0.42) and a moderate and physiologically important effect at 6 mo (MD: −0.8 units; *d* = 0.68; 95% CI: 0.41, 0.88) for Enhanced Protein participants compared with RDA Protein. The Enhanced Protein group increase in SPPB at 3 mo was 1.5 ± 0.2 units, compared with 1.2 ± 0.3 units for the RDA Protein group. At 6 mo, the increase in the Enhanced Protein group was 1.8 ± 0.3 units, compared with 1.0 ± 0.4 for the RDA Protein group. For perspective, a change in SPPB score of 1 or more units is considered physiologically important [29]. As for preservation of FFM (kg), there was a small group effect on the amount lost at 3 mo, with 0.7 ± 0.3 kg lost in the Enhanced Protein group compared with 0.9 ± 0.3 kg lost in the RDA Protein arm (MD: −0.2 kg; *d* = 0.15; 95% CI: <0, 0.33) and a moderate group effect at 6 mo; preservation of FFM was better in the Enhanced Protein group, who lost 0.8 ± 0.3 kg compared with the RDA Protein arm, which lost 1.5 ± 0.4 kg (MD: −0.7 kg; *d* = 0.48; 95% CI: 0.26, 0.62).

#### Secondary outcomes

##### Body composition and waist circumference

Table 2 shows means, MDs, and between-group effect sizes (Cohen’s *d* with 95% CI) at 3 and 6 mo for Enhanced Protein and RDA Control groups. At 3 mo, there was a moderate group effect on body fat, such that the decrease in both the amount (MD: 2.3 kg; *d* = 0.62; 95% CI: −0.03, 1.27) and percent (MD: 1.3%; *d* = 0.62; 95% CI: −0.33, 1.26, 2.6) of body fat was greater in the Enhanced Protein group than in the RDA Protein group. Body fat loss was 4.7 ± 0.9 kg and 2.4 ± 0.5% for Enhanced Protein and 2.4 ± 0.6 kg and 1.1 ± 0.4% for RDA Protein. There was only a small difference between groups at 6 mo for both amount (MD: 0.8 kg; *d* = 0.17; 95% CI: <0, 0.64) and percent body fat (MD: 0.4%; *d* = 0.13; 95% CI: <0, 0.81). A similar pattern was noted for minimal WC, with a greater reduction in Enhanced Protein (reduction of 4.1 ± 0.8 cm) compared with RDA Protein (reduction of 2.6 ± 0.9 cm) at 3 mo (MD: 1.5 cm; *d* = 0.38; 95% CI: 0.06, 0.60) and little difference in WC by group at 6 mo (MD: −0.1 cm; *d* = 0.03; 95% CI: <0, 0.72).

##### Physical function and other measures

At 3 mo from baseline, there was a moderate group effect for both 6MWT and handgrip strength. The Enhanced Protein group increased the distance walked by 63.3 ± 12.8 m (MD: 29.0 m; *d* = 0.53; 95% CI: <0, 1.19) and increased their handgrip strength by 3.7 ± 1.2 kg (MD: −2.8 kg; *d* = 0.56; 95% CI: 0.21, 0.88), relative to the RDA Protein group, which had increases of 34.4 ± 11.9 m and 0.8 ± 1.0 kg for 6MWT and handgrip strength, respectively. A handgrip effect remained at 6 mo for Enhanced Protein, with an increase of 3.0 ± 1.5 kg compared with an increase of 1.2 ± 1.4 kg in RDA Protein (MD: −1.8; *d* = 0.30; 95% CI: 0.15, 0.35), but there was no group difference for 6MWT at 6 mo. For the 8UG result at 3 mo, there was a small effect for Enhanced Protein relative to RDA Protein (MD: 0.3 s; *d* = 0.18; 95% CI: −0.8, 1.4), with no group difference at 6 mo. Although some individual participants had improvements in HbA1c, the mean blood concentrations of HbA1c for the Enhanced Protein group were 6.0 ± 0.3, 5.9 ± 0.3, and 5.9 ± 0.3 at 0, 3, and 6 mo, respectively. The mean concentrations in the RDA Protein group were 6.0 ± 0.3, 5.9 ± 0.3, and 5.9 ± 0.3 at 0, 3, and 6 mo, respectively, with no difference between groups.

## Discussion

We found that both protein treatments had a beneficial effect on SPPB score. However, in agreement with previous findings in other populations of older adults by our group and others [10,31,32], the Enhanced Protein group had a greater increase in SPPB than the RDA Protein group at both 3 and 6 mo. Both groups had a SPPB benefit probably due to the effects of the exercise treatment, as well as a lessened body burden because of body weight reduction. The higher SPPB effect in the Enhanced Protein group could be attributed to the added anabolic effect of generous high-quality protein balanced throughout the day. The anabolic benefit may be manifesting as FFM preservation, as well as the improved SPPB score and other measures of function (6MWT and handgrip strength). The fact that the percentage of weight lost was more in the Enhanced Protein group at 3 mo (4.7% compared with 3.1%) may also help explain the moderately strong effect of protein we found on reducing body fat and WC relative to RDA Protein. It has been previously reported that a balanced higher protein intake is associated with enhanced loss of body fat during a hypocaloric and physical activity intervention [33].

At the 6-mo time point, the superior SPPB score for the Enhanced Protein group was retained, along with the effects of preservation of FFM and handgrip strength. However, there was no group difference in the amount of body weight lost at this time point. At this point, both arms had achieved a weight loss of >6%, exceeding the 5% amount commonly associated with a variety of beneficial changes [34]. This difference in response time could suggest that the Enhanced Protein regimen accelerates the benefits of a diet and exercise intervention for obesity in older adults, such that it takes less time for functional improvements to be attained. One similar finding was reported in a study of postmenopausal females, namely, a protein effect on preservation of muscle mass at 3 mo but not at 6 mo, hinting at a catch-up effect in the control group [35]. However, assessments of function were not included in that trial, so it is unknown whether function also improved [35].

Our findings of no change in circulating concentrations of HbA1c over time in either treatment arm support our hypothesis that higher protein would have no negative impact on glycemic control, although we would have hoped to see some slight improvements along with weight loss. A similar result was reported in sedentary, postmenopausal females with obesity who were consuming a weight loss diet [35]. As for why HbA1c was unaffected in our trial, there are several possible explanations, including insufficient weight loss or insufficient time for HbA1c concentrations to respond to the weight loss. Because both groups had a mean weight loss >6% at 6 mo and both participated in regular (weekly) exercise, the lack of a response is somewhat unexpected. Given that it takes ∼3 mo to see an HbA1c response, it may be that most participants did not reach the amount of weight loss needed to change circulating HbA1c before the 6-mo time point measurement. This explanation would be supported by a study of Japanese males [36] with a very similar baseline HbA1c (5.6%–6.4%) to our population that found a minimum of 4% to 5% weight loss was required to lower diabetes risk. Additionally, Church et al. [37] found that aerobic exercise alone did not significantly change HbA1c concentrations in a group of sedentary males and females completing a 9-mo exercise intervention. Thus, although there was no sign of lessened impact, we are not able to fully address the question of possible blunting of glycemic control response by higher protein intake from our findings.

### Limitations and strengths

A major limitation of this work is the small participant number, which occurred because of the lengthy interruption of clinical research at our facilities owing to the COVID-19 research shutdown (March 2020–October 2021). We were able to change our approach to analyze the study as a pilot trial, looking at the estimated effects of the 2 treatments, but we acknowledge that the small participant numbers limited the achievement of our original study objectives. A second limitation is that all participants were male and either of Black or White race; thus, the results may not be generalizable to females and races not represented in the study population. Finally, it would have been advantageous to have a more sensitive measure of insulin resistance in the study. The original plan to conduct oral glucose tolerance tests at 0 and 6 mo was disrupted by the COVID-19 shutdown and the resulting logistical difficulties and was not possible to conduct. Despite these concerns, the strengths of the trial include rigorous RCT methodological conduct and a focus on understudied participants, namely, male veterans with obesity and prediabetes. Additionally, the RCT was blocked by race to have equal numbers of Black and White participants in each arm. Thus, the study findings provide important preliminary evidence to encourage further study of higher protein diet and exercise regimens to benefit function in this population.

## Conclusion

Our findings of higher mean group differences for enhanced protein benefits to physical function (SPPB, handgrip) extend the results of past trials with higher protein intakes during the challenge of obesity reduction. The significant effects of the enhanced protein regimen on physical function, along with results favoring less body fat and more lean preservation, found in this pilot trial are in agreement with a growing literature based on generous protein intake during obesity reduction in other groups of older adults. These findings provide preliminary evidence that the regimen can also benefit older males with prediabetes, especially with regard to physical function and body composition.

## Author contributions

The authors’ responsibilities were as follows – CWB and KNPS: designed the study; RJS, CWB, JTW, and KNPS: analyzed data and performed statistical analyses; CWB: drafted the manuscript; CWB and KNPS: had primary responsibility for the final content; and all authors: conducted the research, read, and approved the final manuscript.

## Data availability

Data described in the manuscript, code book, and analytic code will not be made available because participants did not consent to open-access sharing.

## Declaration of Generative AI and AI-assisted technologies in the writing process

The authors declare that no generative AI or AI-assisted technologies were used in the writing of this manuscript.

## Funding

This work was funded by VA Rehabilitation Research and Development grant I01RX002843. KNPS is partially supported by the Claude D. Pepper Older Americans Independence Center at Duke University School of Medicine (P30AG028716), and VA Rehabilitation Research and Development grant I01RX003981. We also received food product donations from Smithfield Foods (pork tenderloins), John Soules Foods (chicken and beef strips), and Muscle Feast (whey protein powder).

## Conflict of interest

CWB has an editorial role with Taylor & Francis Group. KNPS is on the speaker bureau for Abbott Nutrition Health Institute and Nestle; Scientific Advisor for Vivo. CWB and KNPS are guest editors for the special issue but they had no involvement in the peer review of this article and had no access to information regarding its peer review. Full responsibility for the editorial process for this article was assigned to another journal editor. All other authors report no conflicts of interest.
